# Demethyleneberberine Protects against Hepatic Fibrosis in Mice by Modulating NF-κB Signaling

**DOI:** 10.3390/ijms17071036

**Published:** 2016-06-30

**Authors:** Yongchen Wang, Zheng Zhao, Yan Yan, Xiaoyan Qiang, Cuisong Zhou, Ruiyan Li, Huan Chen, Yubin Zhang

**Affiliations:** 1 State Key Laboratory of Natural Medicines, Department of Biochemistry, China Pharmaceutical University, Nanjing 210009, China; yongchenwang@g.ucla.edu (Y.W.); benbobo2012@163.com (Y.Y.); qiangxiaoyan0228@126.com (X.Q.); zhoucuisong2009@163.com (C.Z.); liruiyan_1979@126.com (R.L.); njuchenhuan@163.com (H.C.); 2 Divisions of Molecular Medicine and Cardiology, Departments of Anesthesiology and Medicine, David Geffen School of Medicine at University of California Los Angeles, Los Angeles, CA 90095, USA; 3 Department of Pharmacy, Nanjing First Hospital, Nanjing Medical University, Nanjing 210009, China; zhaozheng0322@126.com; 4 Department of Biochemistry, China Pharmaceutical University, 24 Tongjia Xiang, Nanjing 210009, China

**Keywords:** hepatic fibrosis, hepatic stellate cells, cell apoptosis, Demethyleneberberine, NF-κB

## Abstract

Demethyleneberberine (DMB) is an essential metabolite of Berberine (BBR) in vivo. Recent reports have revealed multiple novel therapeutic applications of BBR. However, the pharmacological activities of DMB remain to be elucidated. This study aimed to demonstrate the hepatoprotective and anti-fibrotic effects of DMB both in vitro and in vivo. Here we showed that DMB protects against thioacetamide (TAA)-induced hepatic fibrosis in mice and exhibits a higher safety profile as compared to BBR. Flow cytometry and Western blotting analysis showed that DMB is able to suppress the activation of hepatic stellate cells (HSCs) and induce cell apoptosis through the nuclear factor-κB (NF-κB) cascade. Immunohistochemical (IHC) and quantitative polymerase chain reaction (qPCR) analysis indicated that DMB also has inhibitory effects on collagen synthesis and is able to increase collagen degradation by blocking the transforming growth factor β 1 (*TGF-β1*)-*Smad* signaling and reducing the expression of matrix metalloproteinases (*MMPs*) and tissue inhibitors of *MMP* (*TIMPs*). These findings indicate that DMB has the potential to attenuate hepatic fibrosis via suppressing HSC activation.

## 1. Introduction

Hepatic fibrosis is a common response to many chronic hepatic diseases [1]. The most important pathogenic feature of hepatic fibrosis is the activation of hepatic stellate cells (HSCs), which causes the subsequent accumulation of extracellular matrix (ECM) proteins in liver [2,3,4]. Collagen types I is the primary component of ECM in fibrotic tissue, while serum hydroxyproline is a marker of collagen content and metabolism [5,6]. The expression of ECM proteases (e.g., matrix metalloproteinases, MMPs) [7,8] and their inhibitors (tissue inhibitors of metalloproteinases, TIMPs) increases during fibrogenesis [7]. ECM also incorporates a range of growth factors, like transforming growth factor-β1 (TGF-β1), to modulate the activation and proliferation of HSCs [9,10]. It is reported that active nuclear factor-κB (NF-κB) plays a pivotal role in preventing apoptosis of activated HSCs [11,12]. Therefore, inhibition of active NF-κB is able to effectively suppress the activation of HSCs [13,14].

Treatment with toxic chemicals is an effective way to induce hepatic fibrosis in animal models. The most commonly used hepatotoxic agents include carbon tetrachloride (CCl_4_), thioacetamide (TAA), d-galactosamine, and lipopolysaccharide [15,16,17]. TAA is a water-soluble toxic substance that can induce acute hepatic injury in mice. The cytotoxicity of TAA in vivo mainly relies on its metabolites, TAA sulfoxide and TAA-S-S-dioxide. In hepatocytes, TAA metabolites covalently bind intracellular molecules, consequently causing oxidative stress and inflammation, and inducing activation of HSCs. Cytochrome P450 2E1 (CYP2E1) is required for bioactivation of TAA which induced hepatotoxicity in mouse model, producing reactive oxygen species (ROS) that damages hepatocytes by lipid peroxidation in cell membranes [18,19,20]. These processes activate HSCs and induce them to secrete collagen fibers [21,22]. Therefore, the toxic mechanism of TAA mimics human hepatic fibrosis. Miyazaki et al. [23] established the animal model of liver fibrosis induced by TAA in 1956, which showed high success rate and good repeatability. This model has been widely used in liver fibrosis studies [24,25,26].

Berberine (BBR) is an isoquinoline alkaloid from the traditional Chinese medicine Coptis, which has a widespread pharmacological activities including anti-bacterial, anti-inflammatory, and anti-tumor effects [27,28,29]. Recently, researchers have also investigated the protective effects of BBR against acute and chronic hepatic damage. Xu Sun et al. [30] found that berberine prevents liver fibrosis by inhibiting HSCs proliferation. Our previous study found that BBR has very low oral bioavailability and high toxicity by intravenous injection [31]. Demethyleneberberine (DMB) is a major metabolite of BBR and has lower toxicity than BBR. Previously, we reported that DMB is a natural mitochondria-targeted antioxidant that can inhibit oxidative stress, mitochondrial dysfunction, and steatosis in an alcoholic hepatic disease model [31]. We therefore hypothesize that DMB may also be used for the treatment of hepatic fibrosis based on its hepatoprotective effects and low toxicity [31]. In this study, we demonstrated that DMB has significant hepatoprotective and anti-fibrotic effects against acute hepatic injury and chronic TAA-induced hepatic fibrosis. We also found that DMB is able to suppress NF-κB activation, which indicated that the anti-fibrotic effects of DMB may be exerted through blocking the activation of HSCs.

## 2. Results

### 2.1. Demethyleneberberine (DMB) Alleviates Thioacetamide (TAA)-Induced Acute Hepatic Injury

**Figure 1:**
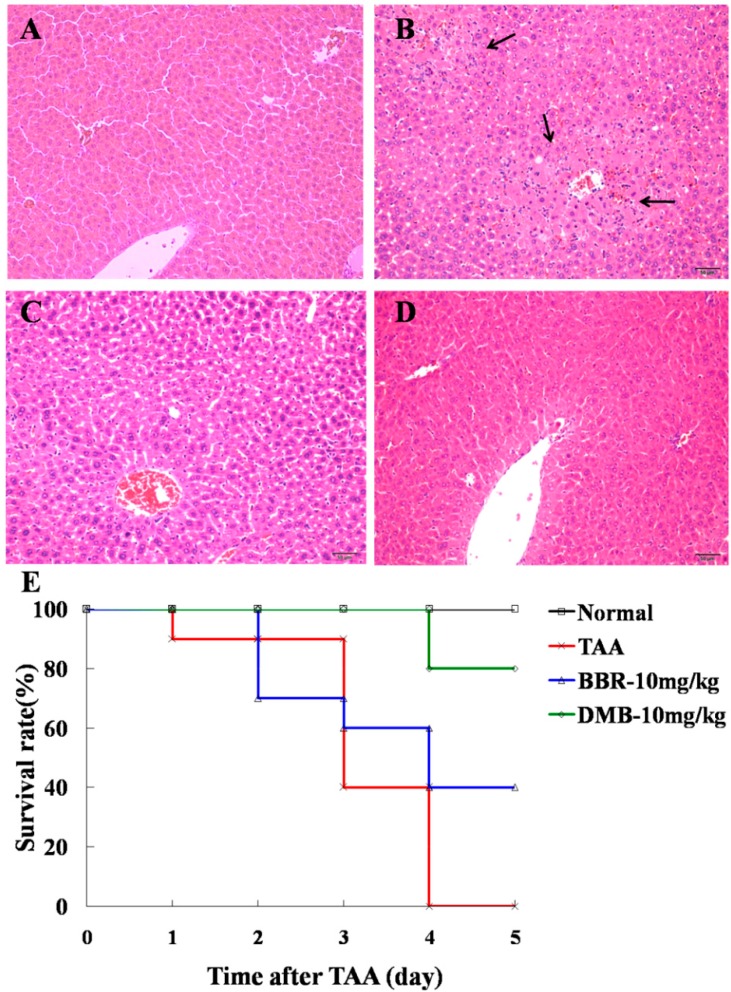
Hematoxylin and eosin (H&E) staining of liver tissue sections from: (**A**) control group; (**B**) thioacetamide (TAA) group; (**C**) berberine (BBR)-treated group (10 mg/kg); and (**D**) demethyleneberberine (DMB)-treated (10 mg/kg) group. Magnification was 200×. Focal necrosis of liver cells and infiltration of inflammatory cells could be observed in the TAA-treated group (arrowheads). Treatment with BBR and DMB remarkably attenuated liver injury. Only a slight eosinophilic change was seen in liver cells without necrosis, and DMB showed better effect than BBR. (**E**) Survival curve of mice for five days after TAA injection.

Eighteen hours after TAA administration (100 mg/kg, intraperitoneal administration (IP)), focal necrosis of hepatic cells and infiltration of inflammatory cells were observed in mice livers by hematoxylin and eosin (H&E) staining (Figure 1). However, treatment with DMB (10 mg/kg, IP) could remarkably alleviate these pathological changes and was more effective than BBR treatment (Figure 1, Table 1). Additionally, the serum levels of alaine aminotrasferase (ALT) and aspartate aminotrasferase (AST) were significantly reduced by DMB treatment (Table 2). These results suggest that DMB may have better protective effects against TAA-induced acute hepatic injury than BBR.

Mice were injected with TAA (250 mg/kg, IP) to induce fulminant hepatic failure. DMB (10 mg/kg, IP) and BBR (10 mg/kg, IP) were administered to mice for five days after TAA challenge. All mice in the model group showed activity reduction; 50% mice in BBR-treated group and 20% mice in DMB-treated group showed activity reduction. As shown in Figure 1E, all animals died from fulminant hepatic failure during the four days after TAA injection. However, treatment with DMB and BBR raised the survival rate to 80% and 40%, respectively. These data show that DMB is able to improve the survival rate of mice with TAA-induced fulminant hepatic failure as compared to BBR.

### 2.2. DMB Attenuates TAA-Induced Hepatic Fibrosis

**Figure 2:**
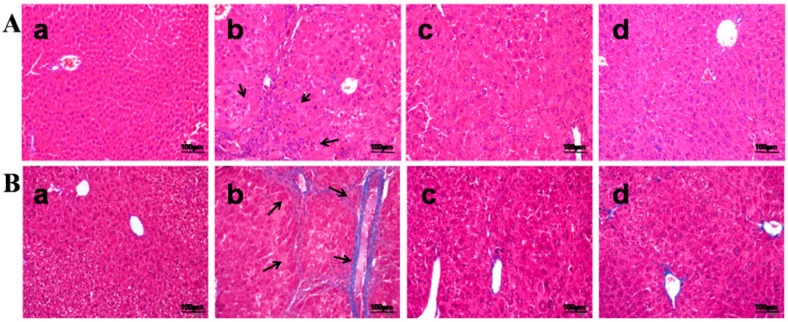
(**A**) H&E-stained liver tissue sections were from: (**a**) control group; (**b**) TAA group; (**c**) low dose DMB-treated group (10 mg/kg); and (**d**) high dose DMB-treated (20 mg/kg) group. Magnification was 200×. Obvious proliferation of collagen fibers with pseudolobuli and infiltration of inflammatory cells could be observed in the TAA model group (arrowheads). In both DMB-treated groups, mild hyperplasia of collagen fibers was presented without pseudolobuli formation and inflammatory cells were significantly reduced; (**B**) Masson’s trichrome staining of liver tissue sections from: (**a**) control group; (**b**) TAA group; (**c**) low dose DMB-treated group (10 mg/kg); and (**d**) high dose DMB-treated (20 mg/kg) group. Magnification was 200×. In the TAA model group, live collagens were widely distributed from the central vein to the surrounding areas (arrowheads). The fibers were coarse and crossed each other forming fibrous septums and obvious pseudolobuli. DMB treatment remarkably attenuated these pathological changes. Collagen fibers were distributed sporadically surrounding the portal area without formation of pseudolobuli.

Mice were injected with TAA three times per week for 10 weeks to induce significant hepatic fibrosis. H&E staining and Masson’s trichrome-staining showed obvious proliferation of collagen fibers with pseudolobuli and infiltration of inflammatory cells in histological sections (Figure 2B, Table 3). In addition, TAA treatment increased liver-to-body weight ratio and serum ALT and AST levels, as well as decreased serum ALB level (Table 4). In DMB-treated animals, however, the proliferation of collagen fibers was mitigated (Figure 2C); and serum ALT and AST levels were reduced by 48.4% and 47.6%, as well as by 49.3% and 38.7% in both high and low dose DMB-treated groups, respectively. Serum ALB level was increased by 10.4% and 22.5%, respectively, in both groups, as compared with the TAA model group. Furthermore, DMB significantly counteracted TAA-induced elevation of hydroxyproline in the liver (Table 4). These results showed that DMB is able to attenuate TAA-induced hepatic fibrosis in mice.

### 2.3. DMB Inhibits the Expression of Alpha-Smooth Muscle Actin (α-SMA)

**Figure 3:**
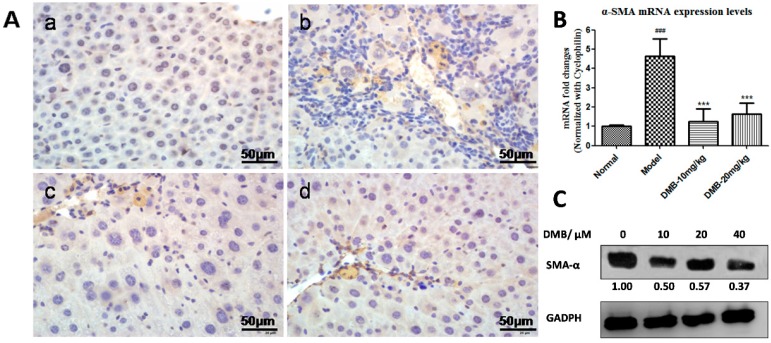
(**A**) Immunohistochemical staining of activated HSCs in the liver tissue sections from: (**a**) control group; (**b**) TAA group; (**c**) low dose DMB-treated group (10 mg/kg); and (**d**) high dose DMB-treated (20 mg/kg) group. Magnification was 200×. α-SMA, a specific marker of activated HSCs, was stained brown. Higher expression of α-SMA was observed in activated HSCs of the TAA-treated liver sections. However, less activated HSCs were found in the DMB-treated sections; (**B**) The expression level of *α-SMA* mRNA in different groups. Cyclophinlin was used as endogenous reference. Values represent means ± SD (*n* = 8). ^###^ *p* < 0.001 compared to the control group; and *** *p* < 0.001 compared to the TAA model group; (**C**) Western blot analysis of α-SMA in HSCs incubated with DMEM or DMB (10, 20, and 40 µM) for 48 h. The level of α-SMA was normalized to GAPDH.

Immunohistochemical (IHC) staining showed no Alpha-Smooth Muscle Actin (α-SMA) expression in the liver sections from control group, whereas significantly marked immune staining was observed around the portal tracts and along fibrous septae in TAA-treated animals. However, α-SMA expression was notably reduced in the DMB-treated group (Figure 3A), indicating the inhibitory effect of DMB on activation of HSCs. quantitative polymerase chain reaction (qPCR) analysis also revealed that *α-SMA* mRNA level was increased in the TAA model group but reduced in the DMB-treated groups (Figure 3B). In cultured HSC-T6 cells, Western blot analysis also showed decreased protein level of α-SMA upon DMB incubation, suggesting that DMB could effectively suppress the activation of HSC-T6 cells (Figure 3C).

### 2.4. DMB Blocks the Growth Factor β 1 (TGF-β1)-Smad Signaling

**Figure 4:**
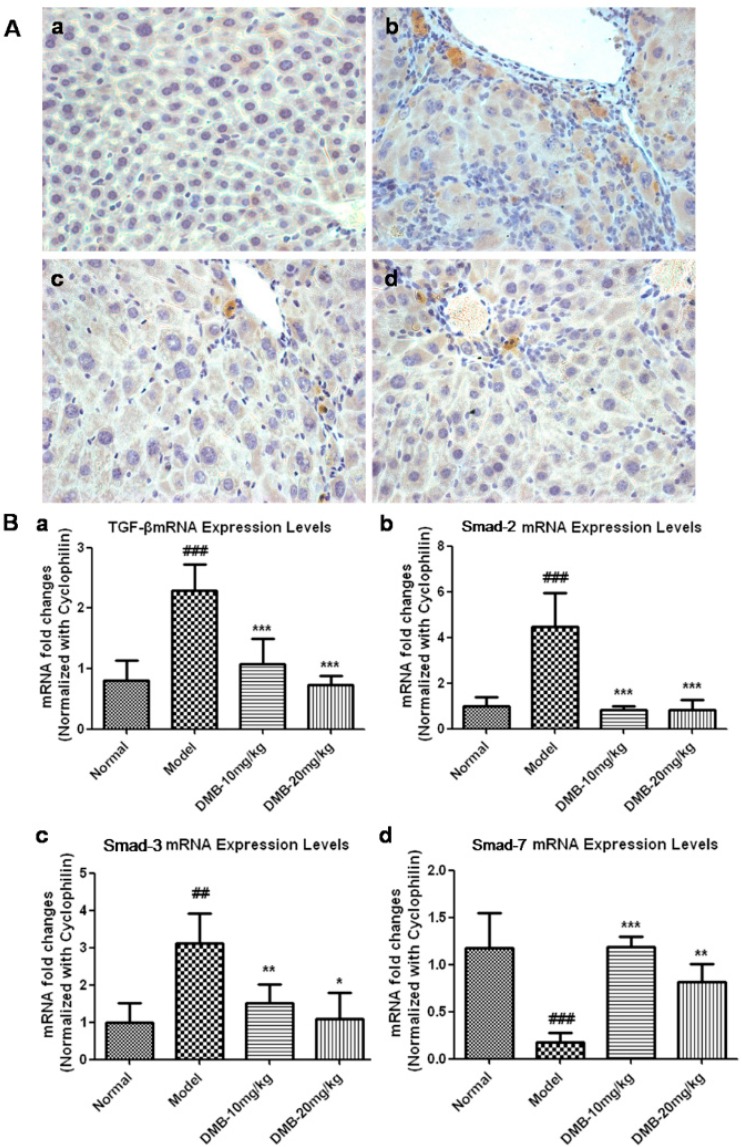
(**A**) IHC staining for activated TGF-β in the liver sections from: (**a**) control group; (**b**) TAA group; (**c**) low dose DMB-treated group (10 mg/kg); and (**d**) high dose DMB-treated (20 mg/kg) group. Magnification was 200×. TGF-β was stained brown and its expression was obviously increased in the central vein, portal area, and fiber septums in the TAA-treated liver sections. However, TGF-β expression was considerably reduced in the DMB-treated groups; (**B**) The mRNA level of: *TGF-β* (**a**); *Smad-2* (**b**); *Smad-3* (**c**); and *Smad-7* (**d**). Cyclophinlin was used as endogenous reference. Values represent means ± SD (*n* = 8). ^##^ *p* < 0.01, and ^###^ *p* < 0.001 compared to the control group; and * *p* < 0.05, ** *p* < 0.01, and *** *p* < 0.001 compared to the TAA model group.

TGF-β1 plays a critical role in the HSCs activation and subsequent acquisition of the fibrogenic function. IHC analysis (Figure 4A) showed that TGF-β1 expression was not observed in the sections from the control group. Nevertheless, histological sections from the TAA-treated group showed a very high TGF-β1 expression level, while this expression was significantly reduced in the DMB-treated groups. qPCR analysis revealed that the mRNA level of TGF-β1 was reduced by 46.7% in the low dose DMB-treated group compared to the TAA group, and returned to the normal level in the high dose DMB-treated group (Figure 4B).

*Smad-2*, *Smad-3*, and *Smad-7* are known to be downstream molecules in the TGF-β1 signaling pathway [32]. As shown in Figure 4B, the mRNA levels of *Smad-2* and *Smad-3* in the TAA model group were increased by 4.4- and 3.1-fold, respectively, in comparison with the control group. In the DMB-treated groups, however, *Smad-2* mRNA was restored to the normal level and *Smad-3* mRNA was reduced by two-fold compared with the TAA model group. On the other hand, the expression of *Smad-7* mRNA was increased by 6.2- and 4.3-fold in the low and high dose DMB-treated groups, respectively, as compared to the model group.

Collectively, these results indicate that DMB can block the *TGF-β1-Smad* signaling cascade and consequently inhibit HSC activation.

### 2.5. DMB Reduces the Expression of Matrix Metalloproteinases (MMPs) and Tissue Inhibitors of MMP (TIMPs)

**Figure 5:**
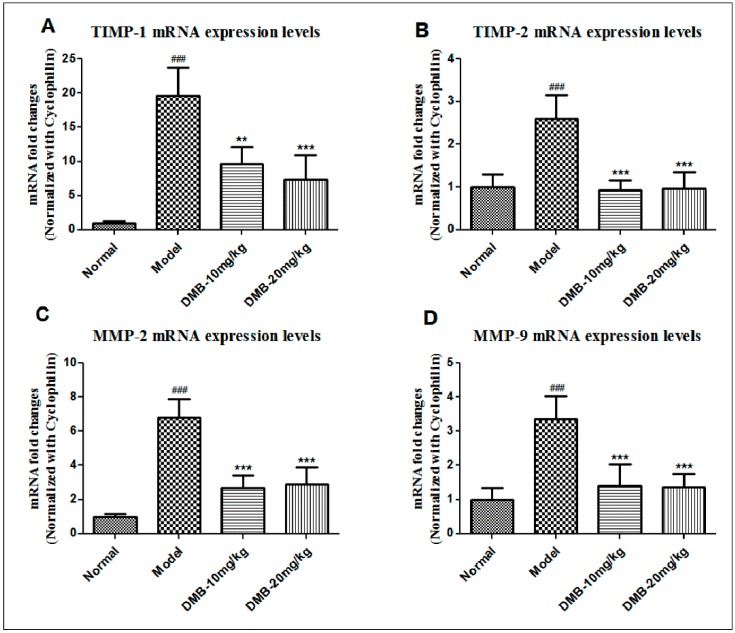
The mRNA level of: *TIMP-1* (**A**); *TIMP-2* (**B**); *MMP-2* (**C**); and *MMP-9* (**D**). Cyclophinlin was used as endogenous reference. Values are presented as means ± SD (*n* = 8). ^###^ *p* < 0.001 compared to the control group; and ** *p* < 0.01, and *** *p* < 0.001 compared to the TAA model group.

The expression of *TIMP*s and *MMP*s increases during chronic fibrosis, while *TIMP-1* and *TIMP-2* are the specific inhibitor of *MMP-9* and *MMP-2*, respectively. As shown in Figure 5, the mRNA levels of *MMP-2*, *MMP-9*, *TIMP-1*, and *TIMP-2* were much higher in the TAA model group than in the control group. Nevertheless, in the DMB-treated group, *TIMP-1* mRNA level was decreased by 20% as compared with the TAA model group, and *TIMP-2* mRNA was resumed to the normal level. These results showed that DMB inhibits the expression of *TIMP*s, thus promoting the degradation of collagen. Additionally, *MMP-2* is known to be expressed and released from activated HSCs, while *MMP-9* is from inflammatory cells. We found that *MMP-2* and *MMP-9* mRNA levels were increased by seven- and 3.5-fold, respectively, in the TAA model group, in comparison with the control group. However, DMB treatment could reduce *MMP-2* and *MMP-9* mRNA levels upon TAA exposure. These results revealed that DMB can block HSC activation as well as reduce the infiltration of inflammatory cells through suppressing mRNA expression of *MMP* and *TIMP*.

### 2.6. DMB Promotes Apoptosis of Hepatic Stellate Cells (HSCs) via Suppressing Active Nuclear Factor-κB (NF-κB) Activation

**Figure 6:**
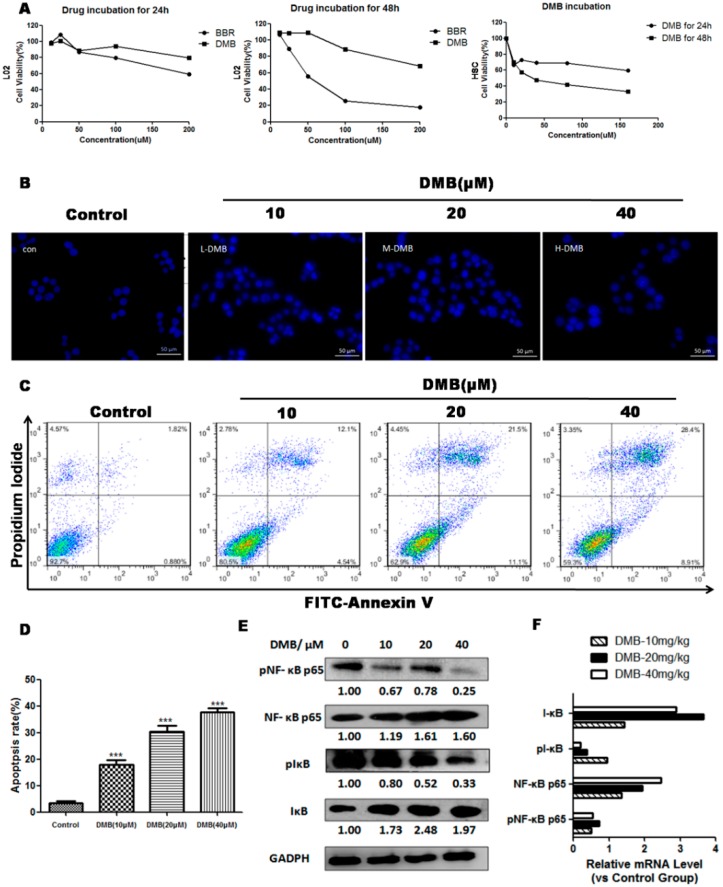
(**A**) After serum-starvation for 24 h, L02 and HSC-T6 cells were exposed to DMB or BBR for 24 and 48 h, and cell viability was then assayed using the 3-(4,5-dimethylthiazol-2-yl)-2,5-diphenyltetrazolium bromide (MTT) method; (**B**) Cell morphology under fluorescence microscopy by Hoechst 33258 staining in HSCs incubated with DMEM or DMB for 48 h. Panels represent the control group and the groups exposed to 10, 20, and 40 µM of DMB, respectively. The DMB-treated HSC-T6 cells exhibited morphological changes of nuclei (**C**) Flow cytometry analysis by Annexin V-Fluorescein isothiocyanate/propidium iodide (FITC/PI) double-staining in HSC-T6 cells incubated with DMEM or difference doses of DMB (10, 20, and 40 µM) for 48 h; (**D**) The percentages of cells at different stages (viable, apoptotic, and necrotic) in HSCs incubated with DMEM or DMB for 48 h; (**E**) Western blot analysis of apoptosis-related proteins in HSCs incubated with DMEM or DMB (10, 20, and 40 µM) for 48 h. The level of p-NF-κB P65 was normalized to its unphosphorylated form, while the levels of IκBα, and p-IκBα were normalized to GAPDH; (**F**) qPCR analysis of related gene levels in DMB (10, 20, 40 mg/kg) treated HSCs, compared to untreated HSCs. Representative blots were from three independent experiments. Values represent means ± SD (*n* = 3). *** *p* <0.001 compared to the control group.

HSC-T6 cells were incubated with six concentrations of DMB (0, 10, 20, 40, 80, and 160 µmol/L) for 24 and 48 h. 3-(4,5-dimethylthiazol-2-yl)-2,5-diphenyltetrazolium bromide (MTT) assay showed that DMB significantly inhibit proliferation of HSCs in a concentration dependent manner (Figure 6A). The IC_50_ value of DMB for HSC-T6 cells at 48 h was 36.7 µmol/L. Therefore, we chose 0, 10, 20 and 40 µmol/L for further experiments.

Hoechst 33258 staining demonstrated that the nuclei morphology of HSC-T6 was damaged and exhibited nuclear shrinkage, chromatin condensation, and formation of apoptotic bodies in cells treated by 10–40 µmol/L of DMB (Figure 6B). To further quantify the apoptosis induced by DMB, we performed flow cytometry by Fluorescein isothiocyanate (FITC)-labeled annexin V/propidium iodide (PI) staining (Figure 6C,D). The results exhibited that DMB could induce a significant dose-dependent increase in apoptosis of HSC-T6 when compared with the control group. In addition, we treated normal hepatocyte L02 using DMB, but little apoptosis was detected, which suggested that DMB triggers apoptosis specifically in HSCs (Figure 6A).

To investigate the molecular mechanism of DMB-triggered apoptosis in HSCs, we detected phosphorylation of NF-κB p65 and IκBα in HSCs. Western blot and related mRNA expression analysis demonstrated that DMB markedly suppressed phosphorylation of NF-κB p65, while there was no change in total NF-κB p65 abundance (Figure 6E,F). These findings confirmed that DMB decreased the translocation of NF-κB p65 from the cytoplasm to the nucleus. Simultaneously, p-IκBα expression level was decreased upon DMB incubation, but the level of total IκBα was elevated, indicating that DMB could inhibit phosphorylation and degradation of IκBα. These results suggested that DMB induces apoptosis in HSCs, at least in part, via suppressing NF-κB activation.

## 3. Discussion

Hepatic fibrosis is theoretically a reversible disease, but few drugs can be used in clinically used to reverse its pathological process. BBR has been reported having a wide variety of therapeutic effects, including the protective activity against chronic hepatic fibrosis [33]. However, BBR has very low oral bioavailability and high toxicity by intravenous injection [34]. DMB has been identified as a major metabolite of BBR and an ingredient of Cotex Phellodendri Chinensis. Our study demonstrated that LD_50_ of DMB is 30 mg/kg (intravenous injection, IV) (data not shown here), which is greatly higher than that of BBR (9.0 mg/kg) [35]. Cell viability assay further verifies that the toxicity of DMB is significantly lower than that of BBR. In this study, we found that most of animals in the BBR-treated group died due to high toxicity of BBR on long-term intraperitoneal injection (data not shown). All of the above indicate that DMB has lower toxicity and higher safety than BBR.

For the animal studies, first we induced the acute hepatic injury to evaluate the liver protective effects of DMB and BBR against low dose TAA (100 mg/kg) and we found DMB showed better protective effects than BBR. To validate this conclusion, fulminant hepatic failure experiment was then conducted to evaluate the toxicity tolerance of mice on high dose TAA (250 mg/kg) after treatment with BBR or DMB. The final survival rate of mice in the DMB-treated group was also much higher than that in the BBR-treated group. The TAA doses are based on other reported studies [36,37]. Through these two experiments, we confirmed that DMB has significantly more protective effects. Moreover, we provide evidence for the subsequent chronic hepatic fibrosis study. We speculate that transforming to DMB by CYP450 is necessary for BBR to antagonize the toxicity of TAA. When the liver is severely impaired by TAA, the amount of DMB generated from BBR metabolism decreases, followed by the reduced protective effects. Higher protective effects and lower toxicity make us believe that DMB may serve as a surrogate of BBR with functions that are both unique from and complementary to BBR.

We investigated the molecular mechanism underlying the anti-fibrotic effect of DMB treatment. In this study, HSC activation can be considered a core mechanism. IHC and cell culture results indicated that after DMB treatment, the protein expression of α-SMA, which is considered as a marker of HSCs activation and liver fibrosis [38,39,40], was remarkably reduced. We also found that DMB incubation could effectively suppress the proliferation of HSC-T6 and promote cell apoptosis. Various researchers have pointed out that HSC activation can be inhibited by blocking the NF-κB signaling pathway, which plays an important role in apoptosis of HSC-T6 [41,42,43]. Western blot analysis showed that DMB incubation significantly inhibited NF-κB activation. Some other literature reported NF-κB activity is not required for cell activation [44]; however, our results demonstrated that the NF-κB signaling pathway does affect the apoptosis of HSC-T6.

Kupffer cells are stimulated by damaged cells to secrete TGF-β1. Previous research showed that hepatic fibrosis can be developed intransgenic mice overexpressing TGF-β1, while silencing of TGF-β1 gene could significantly reduce hepatic fibrogenesis [45]. In our study, TGF-β1 expression in the DMB-treated group was significantly reduced when compared with the TAA model group. Results of qPCR displayed that mRNA levels of the genes closely related to collagen synthesis and degradation were all down-regulated. Based on these results, we assume that the main mechanism involved in anti-fibrotic effect of DMB is to suppress the activation of HSCs, as well as reduce the expression of TGF-β1 and TIMPs, which consequently decreases ECM synthesis and promotes collagen degradation.

Liver fibrosis occurs in most types of chronic liver diseases, inhibiting the activity of HSCs should be the most effective treatment strategy. This study is the first time that demonstrates the anti-liver fibrosis effect of DMB by suppressing HSCs activation via modulating the NF-κB signaling. However, we will use transgenic mice to validate our conclusions in the future study and more detailed molecular mechanism will be further investigated.

## 4. Materials and Methods

### 4.1. Reagents

TAA from Sagon Biotech (Shanghai, China) was dissolved in sterile saline solution. DMB hydrochloride (purity > 98%) was synthesized by our laboratory, dissolved in dimethyl sulfoxide (DMSO), and diluted with sterile saline solution to a final concentration (DMSO = 0.1%) for animal experiments [31]. Antibodies were purchased from Cell Signaling Technology (Danvers, MA, USA). Dulbecco’s modified Eagle medium (DMEM), fetal bovine serum (FBS) and calf serum (CS) were purchased from Gibco (Gland Island, NY, USA). Hoechst 33258 was purchased from Sigma (St. Louis, MO, USA) and the Annexin V-Fluorescein isothiocyanate (FITC) Apoptosis Detection Kit I was purchased from Becton Dickinson (SanDiego, CA, USA). All other reagents were analytical or biological grade.

### 4.2. Cell Culture and Treatments

Rat hepatic stellate-T6 cells (HSC-T6, KeyGEN, Nanjing, China) and human hepatic L02 cells (KeyGEN) were cultured in DMEM containing 10% CS or 10% FBS, 1% glutamine, 100,000 IU/L penicillin, and 100 mg/L streptomycin at 37 °C with 5% CO_2_ in air. Cell viability was determined by 3-(4,5-dimethylthiazol-2-yl)-2,5-diphenyltetrazolium bromide (MTT) assay after 24 and 48 h incubation with 0–200 µmol/L DMB or BBR. For Western blot or quantitative PCR (qPCR) analysis, cells were incubated with different concentrations of DMB (0–40 µmol/L) for 48 h and then collected for subsequent assays. The selection of drug concentrations was based on our earlier studies [31,46]. Cell morphology was observed using Hoechst 33258 staining and photographed by a Zeiss fluorescence microscope (Zeiss Vert. A1, Jena, Germany).

Cell apoptosis analysis was performed by flow cytometry (BD Biosciences, San Jose, CA, USA). After being treated by various doses of DMB (0, 10, 20, and 40 µmol/L) for 48 h, cells were stained by propidium iodide (PI) and the Annexin V-FITC Apoptosis Detection Kit I according to manufacturer’s protocol. The percentage of Annexin V-positive cells was evaluated using FlowJo software (Tree Star, San Carlos, CA, USA).

### 4.3. Animal Experiments

Male ICR mice (24–26 g) were purchased from the Laboratory Animal Center of Yangzhou University (Yangzhou, China) and kept in a temperature- and humidity-controlled animal room (25 ± 2 °C, 50% ± 10% relative humidity) with 12 h light/dark cycles. Animals were fed with standard diet and water. All procedures were approved by the Institutional Animal Care and Use Committee (212906, 07/01/2012-06/30/2017) of China Pharmaceutical University (Nanjing, China) and adhered to the Guidelines of Jiangsu Province for animal experimentation.

In the experiment of acute hepatic injury, 24 mice were randomized into four groups: the vehicle control group (sterile saline solution containing 0.1% DMSO, IP), the TAA model group (TAA, 100 mg/kg, IP), the BBR-treated group (100 mg/kg TAA + 10 mg/kg BBR, IP), and the DMB-treated group (100 mg/kg TAA + 10 mg/kg DMB, IP). BBR or DMB were treated at 8 and 16 h after TAA administration. Animals were sacrificed 18 h following TAA administration. Mice were anesthetized with isoflurane before sacrifice and removing one eyeball to collect the blood. Mice did not wake up during this process and bled to death. Liver sections were collected in formalin-PBS for hematoxylin and eosin H&E staining and other studies. In the experiment of fulminant hepatic failure, the dosage of TAA was increased to 250 mg/kg. A total of 40 mice were randomly divided into four groups: the vehicle control group (sterile saline solution containing 0.1% DMSO, IP), the TAA model group (TAA, 250 mg/kg, IP), the DMB-treated group (250 mg/kg TAA + 10 mg/kg DMB, IP), and the BBR-treated group (250 mg/kg TAA + 10 mg/kg BBR, IP). The experiment last for five days, survival, activity, and mental condition of the animals were recorded every day.

In the experiment of chronic hepatic injury, 32 mice were randomly assigned into four groups: the vehicle control group (sterile saline solution containing 0.1% DMSO, IP), the TAA model group, the low dose DMB-treated group (10 mg/kg DMB, IP), the high dose DMB-treated group (20 mg/kg DMB, IP). TAA was intraperitoneally injected to animals three times a week for ten weeks (100 mg/kg for the first two weeks and 200 mg/kg for the next eight weeks). Both DMB-treated groups were administered daily. The animals were sacrificed 48 h after the final injection of TAA. Tissue and blood samples were harvested as described before for subsequent analyses.

### 4.4. Pathological Evaluation

H&E staining was assessed for steatosis, inflammation, and necrosis. Liver pathology scores were described by Nanji et al [47]. The following scores were assigned for steatosis (the fat percentage in liver cells): 1, <25%; 2, <50%; 3, <75%; or 4, >75%; and inflammation and necrosis: 1:1 focus per low-power field; or 2:2 or more focus per low-power field. Both scores were calculated as a total score for each liver to show the severity of histological abnormality.

Fibrosis was graded according to the method of Ruwart et al. [48]: grade 0, normal liver; grade 1, increase of collagen but no formation of septa; grade 2, incomplete septa from the portal tract to the central vein; grade 3, complete but thin septa , which interconnected with each other and divide the parenchyma into separate fragments; and grade 4, as grade 3 but with thick septa (complete cirrhosis).

### 4.5. Biochemical Assay

Blood (500 µL) was withdrawn from the orbital sinus to an Eppendorf tube and incubated at room temperature for 1 h. Then, the serum was separated by centrifugation (6000× *g* for 5 min). Serum ALT and AST were determined using commercial kits from Spectrum Diagmostics (Jiancheng Biogineering Institute, Nanjing, China).

Hydroxyproline was detected by alkaline hydrolysis using the commercial kit (Jiancheng Biogineering Institute). The absorbance of the colored product was recorded at 550 nm, and the amount of hydroxyproline was expressed as µg/g wet tissue.

### 4.6. Immunohistochemical Analysis

For immunohistochemical (IHC) analysis, 5-µm liver sections were deparaffinized, rehydrated, and incubated in 3% H_2_O_2_ for 20 min to quench endogenous peroxidase activity. After blocking nonspecific staining with normal goat serum, the sections were incubated with polyclonal antibodies against TGF-β1 (1:100, Bioworld, St. Louis, MO, USA) and α-SMA (1:100, Bioworld, St. Louis, MO, USA) overnight at 4 °C. After being washed by PBS, the sections were incubated with goat anti-rabbit antibody at room temperature for 50 min. The antibody binding sites were visualized using 3,30-diaminobenzidine tetrahydrochloride (DAB) and lightly counter-stained with hematoxylin.

### 4.7. qPCR and Western Blot Analysis

Total RNA was extracted from mouse liver using Trizol reagent (Invitrogen, Carlsbad, CA, USA). cDNA was reverse transcribed from 5 µg of total RNA using oligo (dT) primers and M-MLV reverse transcriptase (Promega, Fitchburg, WI, USA). The expression of mRNA for *TGF-β1*, *α-SMA*, *collagen α-1*, *Smad-2, Smad-3*, *Smad-7*, *MMP-2*, *MMP-9*, *TIMP-1*, and *TIMP-2* was analyzed by qPCR with SYBR Green MasterMix (ROX) (Roche, Upper Bavaria, Germany) in a StepOne Plus System (Applied Biosystems, Foster, CA, USA). Assays were repeated twice and the relative abundance of mRNA was normalized to cyclophinlin mRNA using the comparative *C*_t_ method (^ΔΔ^*C*_t_) [49]. The primer sequences used in this study are listed in Table 5.

For Western blot analysis, cells were harvested and sonicated in ice-cold lysis buffer (RIPA) containing protease inhibitor cocktail and phosphatase inhibitor (PhosSTOP. Roche, Switzerland). Lysates were centrifuged at 10,000× *g*, 4 °C for 20 min and the supernatant was collected for protein concentration determination using the bicinchoninic acid (BCA) method. 100 µg of proteins were subjected to sodium dodecyl sulphate-polyacrylamide gel electrophoresis (SDS-PAGE) and transferred to polyvinylidene fluoride (PVDF) membranes (Millipore, Billerica, MA, USA). The membranes were blocked using TBST buffer containing 5% bull serum albumin (BSA) at room temperature for 1 h, and then incubated with primary antibodies and HRP-labeled secondary antibodies. After being washed three times by TBST, the membranes were detected using an enhanced chemiluminescence (ECL) kit (Millipore, Billerica, MA, USA). The expression levels of NF-κB p65, p-NF-κBp65, IκBα, p-IκBα, and α-SMA were quantified by ImageJ software with normalization to GAPDH.

### 4.8. Statistical Analysis

All analyses were performed with Statistical Package for Social Sciences version 16.0 (SPSS, Chicago, IL, USA). Results were expressed as mean ± standard deviation (SD). Statistical significance was determined by one-way analysis of variance (ANOVA). A *p* value < 0.05 was considered statistically significant difference.

## 5. Conclusions

DMB is a major metabolite of BBR in vivo. In our study, results of cell and animal experiments demonstrated DMB has a potential anti-fibrotic effect and higher safety profile as compared to BBR. Data of in vitro and in vivo assays suggested that the main underlying mechanism was to suppress the activation of HSCs, as well as reduce the expression of TGF-β1 and TIMPs, which consequently decreases ECM synthesis and promotes collagen degradation. All of these findings raise the possibility of DMB as a potential and beneficial medication for hepatic fibrosis.

## Figures and Tables

**Table 1:** Treatment effects of demethyleneberberine (DMB) on liver pathological changes in mice with thioacetamide (TAA)-induced *a* Values represent the number of mice rated with a given level of pathological changes.

Group	Liver Pathological Evaluation *^a^*					Average *^b^*
	0	1	2	3	4	
Normal	6	0	0	0	0	0
TAA	0	0	1	2	3	3.3 ± 0.5 ^###^
TAA + BBR (10 mg/kg)	0	0	6	0	0	2.0 ± 0.0 **
TAA + DMB (10 mg/kg)	0	3	3	0	0	1.5 ± 0.5 ***

*^a^* Values represent the number of mice rated with a given level of pathological changes; *^b^* Values represent mean ± SD (*n* = 6); ^###^ *p* < 0.001 compared to normal group; and ** *p* < 0.01, and *** *p* < 0.001 compared to TAA group.

**Table 2:** Treatment effects of DMB on serum alaine aminotrasferase (ALT) and aspartate aminotrasferase (AST) levels in mice with TAA-induced acute liver injury.

Group	ALT (IU/L)	AST (IU/L)
Normal	18.0 ± 2.1	41.8 ± 1.8
TAA	3063.9 ± 161.6 ^###^	208.0 ± 20.6 ^###^
TAA + BBR (10 mg/kg)	1943.6 ± 372.3 ***	123.8 ± 21.2 ***
TAA + DMB (10 mg/kg)	1661.8 ± 78.3 ***	121.9 ± 10.4 ***

Values represent means ± SD (*n* = 6). ^###^ *p* < 0.001 compared to normal group; and *** *p* < 0.001, compared to TAA group. BBR, berberine.

**Table 3:** The pathological score of TAA-induced liver fibrosis in mice.

Group	Severity Score of Liver Fibrosis *^a^*					Average *^b^*
	0	1	2	3	4	
Normal	8	0	0	0	0	0
TAA	0	0	0	2	6	3.8 ± 0.5 ^###^
DMB-10 mg/kg	0	4	4	0	0	1.5 ± 0.5 ***
DMB-20 mg/kg	0	5	3	0	0	1.4 ± 0.5 ***

*^a^* Data represent the number of mice rated with a given level of hepatic fibrosis: (0) normal; (1) very slight; (2) slight; (3) moderate; and (4) severe; *^b^* The individual severity rates in mice were expressed as mean ± SD (*n* = 8). ^###^ *p* < 0.001 compared to normal group; and *** *p* < 0.001 compared to TAA group.

**Table 4:** Effects of DMB on Hypdroxyproline, serum ALT, AST, ALB and liver-to-body weight ratio serum in mice with TAA-induced liver fibrosis.

Group	Hypdroxyproline (µg/g Wet Weight)	ALT (IU/L)	AST (IU/L)	ALB (g/L)	Body Weight (g)	Liver Weight (g)	Liver/Body Weight Ratio (%)
Normal	5.2 ± 13.8	21.4 ± 5.7	29.9 ± 3.9	42.9 ± 3.0	39.9 ± 3.5	1.6 ± 0.2	4.4 ± 0.4
TAA	225.9 ± 26.6 ^###^	151.6 ± 14.8 ^###^	69.3 ± 7.7 ^###^	34.6 ± 1.6 ^###^	34.7 ± 3.1 ^##^	2.3 ± 0.3 ^###^	6.8 ± 0.5 ^###^
DMB-10 mg/kg	119.4 ± 20.6 ***	78.3 ± 7.3 ***	36.3 ± 7.4 ***	38.2 ± 1.7 *	36.5 ± 2.2	1.8 ± 0.3 **	5.3 ± 0.3 ***
DMB-20 mg/kg	117.3 ± 11.8 ***	76.9 ± 9.9 ***	42.5 ± 6.4 ***	42.4 ± 1.7 ***	36.4 ± 2.3	1.7 ± 0.1 ***	5.2 ± 0.3 ***

Values represent means ± SD (*n* = 8). The bodyweight of TAA group has significant difference compared with control group (^##^ *p* < 0.01), there are no significant difference between TAA and DMB-treated groups; ^###^ *p* < 0.001 compared to normal group; and * *p* < 0.05, ** *p* < 0.01, and *** *p* < 0.001 compared to TAA group.

**Table 5:** Primer sequences for Polymerase Chain Reaction (PCR) analysis in this study.

Gene	Forward Primer	Reverse Primer
*α-SMA*	5′-TGACCCAGATTATGTTTGAGACC-3′	5′-CCAGAGTCCAGCACAATACCA-3′
*COL1-α1*	5′-GAGCGGAGAGTACTGGATCG-3′	5′-GTTCGGGCTGATGTACCAGT-3′
*TGF-β1*	5′-CGCCATCTATGAGAAAACC-3′	5′-GTAACGCCAGGAATTGT-3′
*TIMP-1*	5′-GGAAAGCCTCTGTGGATATG-3′	5′-AACAGGGAAACACTGTGC-3′
*TIMP-2*	5′-TTCCGGGAATGACATCTATGG-3′	5′-GGGCCGTGTAGATAAACTCGAT-3′
*MMP-2*	5′-GCTGATACTGACACTGGTACTG-3′	5′-CAATCTTTTCTGGGAGCTC-3′
*MMP-9*	5′-GGAACTCACACGACATCTTCCA-3′	5′-GAAACTCACACGCCAGAAGAATTT-3′
*Smad-2*	5′-ATGTCGTCCATCTTGCCATT-3′	5′-ATTCTGCTCTCCACCACCTG-3′
*Smad-3*	5′-GTAGAGACGCCAGTTCTACC-3′	5′-GGTTTGGAGAACCTGCGTCCAT-3′
*Smad-7*	5′-CAAGAGGCTGTGTTGCTGTG-3′	5′-TGGGTATCTGGAGTAAGGAGGA-3′
*Cyclophilin*	5′-CCATCG TGTCATCAAGGACTTCAT-3′	5′-CTTGCCATCCAGCCAGGAGGTCTT-3′

## References

[1] Wu X., Zhang F., Xiong X., Lu C., Lian N., Lu Y., Zheng S. (2015). Tetramethylpyrazine reduces inflammation in liver fibrosis and inhibits inflammatory cytokine expression in hepatic stellate cells by modulating NLRP3 inflammasome pathway. IUBMB Life.

[2] Hautekeete M.L., Geerts A. (1997). The hepatic stellate (Ito) cell: Its role in human liver disease. Virchows Arch..

[3] Li D., Friedman S.L. (1999). Liver fibrogenesis and the role of hepatic stellate cells: New insights and prospects for therapy. J. Gastroen. Hepatol..

[4] Suhaimi N.A.M., Zhuo L. (2012). Imidazolium salt attenuates thioacetamide-induced liver fibrosis in mice by modulating inflammation and oxidative stress. Dig. Liver Dis..

[5] Di Lullo G.A., Sweeney S.M., Korkko J., Ala-Kokko L., San Antonio J.D. (2002). Mapping the ligand-binding sites and disease-associated mutations on the most abundant protein in the human, type I collagen. J. Biol. Chem..

[6] Kurikawa N., Suga M., Kuroda S., Yamada K., Ishikawa H. (2003). An angiotensin II type 1 receptor antagonist, olmesartan medoxomil, improves experimental liver fibrosis by suppression of proliferation and collagen synthesis in activated hepatic stellate cells. Br. J. Pharmacol..

[7] Hemmann S., Graf J., Roderfeld M., Roeb E. (2007). Expression of MMPs and TIMPs in liver fibrosis—A systematic review with special emphasis on anti-fibrotic strategies. J. Hepatol..

[8] Stravitz R.T. (2008). Critical management decisions in patients with acute liver failure. Chest.

[9] Lin J.G., Chen A.P. (2008). Activation of peroxisome proliferator-activated receptor-γ by curcumin blocks the signaling pathways for PDGF and EGF in hepatic stellate cells. Lab. Investig..

[10] Li J., Li J., Li S., He B., Mi Y.L., Cao H.C., Zhang C.Q., Li L.J. (2012). Ameliorative effect of grape seed proanthocyanidin extract on thioacetamide-induced mouse hepatic fibrosis. Toxicol. Lett..

[11] Vasiliou V., Lee J., Pappa A., Petersen D.R. (2000). Involvement of p65 in the regulation of NF-κB in rat hepatic stellate cells during cirrhosis. Biochem. Biophys. Res. Commun..

[12] He X., Pu G., Tang R., Zhang D., Pan W. (2014). Activation of nuclear factor κB in the hepatic stellate cells of mice with schistosomiasis japonica. PLoS ONE.

[13] Barnes P.J., Larin M. (1997). Mechanisms of disease—Nuclear factor-κB—A pivotal transcription factor in chronic inflammatory diseases. N. Engl. J. Med..

[14] Benfield C.T.O., Mansur D.S., McCoy L.E., Ferguson B.J., Bahar M.W., Oldring A.P., Grimes J.M., Stuart D.I., Graham S.C., Smith G.L. (2011). Mapping the I κB kinase β (IKK β)-binding interface of the B14 protein, a vaccinia virus inhibitor of IKK β-mediated activation of nuclear factor κB. J. Biol. Chem..

[15] Rousar T., Kucera O., Krivakova P., Lotkova H., Kandar R., Muzakova V., Cervinkova Z. (2009). Evaluation of oxidative status in acetaminophen-treated rat hepatocytes in culture. Physiol. Res..

[16] Yamamoto K., Sasakawa Y., Nakaoka F., Nakao M., Nakamura M., Kominami A., Abe M., Fukuhama C., Kagawa K. (2011). Effect of globin digest on the liver injury and hepatic gene expression profile in galactosamine-induced liver injury in SD rats. Life Sci..

[17] Huang H.L., Wang Y.J., Zhang Q.Y., Liu B., Wang F.Y., Li J.J., Zhu R.Z. (2012). Hepatoprotective effects of baicalein against CCl_4_-induced acute liver injury in mice. World J. Gastroenterol..

[18] Fitzhugh O.G., Nelson A.A. (1948). Liver tumours in rats fed thiourea or thioacetamide. Science.

[19] Chilakapati J., Shankar K., Korrapati M.C., Hill R.A., Mehendale H.M. (2005). Saturation toxicokinetics of thioacetamide: Role in initiation of liver injury. Drug Metab. Dispos..

[20] Kang J.S., Wanibuchi H., Morimura K., Wongpoomchai R., Chusiri Y., Gonzalez F.J., Fukushima S. (2008). Role of CYP2E1 in thioacetamide-induced mouse hepatotoxicity. Toxicol. Appl. Pharm..

[21] DiezFernandez C., Sanz N., Cascales M. (1996). Intracellular calcium concentration impairment in hepatocytes from thioacetamide-treated rats. Implications for the activity of Ca^2+^-dependent enzymes. J. Hepatol..

[22] Stankova P., Kucera O., Lotkova H., Rousar T., Endlicher R., Cervinkova Z. (2010). The toxic effect of thioacetamide on rat liver in vitro. Toxicol. In Vitro.

[23] Miyazaki H., Wada A., Takayanagi H. (1956). Histological studies of liver cirrhosis in white rats by thioacetamide feeding. Gan.

[24] Palacios R.S., Roderfeld M., Hemmann S., Rath T., Atanasova S., Tschuschner A., Gressner O.A., Weiskirchen R., Graf J., Roeb E. (2008). Activation of hepatic stellate cells is associated with cytokine expression in thioacetamide-induced hepatic fibrosis in mice. Lab. Investig..

[25] De David C., Rodrigues G., Bona S., Meurer L., Gonzalez-Gallego J., Tunon M.J., Marroni N.P. (2011). Role of quercetin in preventing thioacetamide-induced liver injury in rats. Toxicol. Pathol..

[26] Shaker M.E., Shiha G.E., Ibrahim T.M. (2011). Comparison of early treatment with low doses of nilotinib, imatinib and a clinically relevant dose of silymarin in thioacetamide-induced liver fibrosis. Eur. J. Pharmacol..

[27] Jiang Q., Liu P.Q., Wu X.Q., Liu W.H., Shen X.Y., Lan T.A., Xu S.W., Peng J., Xie X., Huang H.Q. (2011). Berberine attenuates lipopolysaccharide-induced extracelluar matrix accumulation and inflammation in rat mesangial cells: Involvement of NF-κB signaling pathway. Mol. Cell. Endocrinol..

[28] Li J., Cao B., Liu X.C., Fu X.Q., Xiong Z.G., Chen L., Sartor O., Dong Y., Zhang H.T. (2011). Berberine suppresses androgen receptor signaling in prostate cancer. Mol. Cancer Ther..

[29] Wu K., Yang Q.J., Mu Y.Q., Zhou L.Y., Liu Y.Z., Zhou Q.X., He B.C. (2012). Berberine inhibits the proliferation of colon cancer cells by inactivating Wnt/β-catenin signaling. Int. J. Oncol..

[30] Sun X., Zhang X.D., Hu H., Lu Y.N., Chen J., Yasuda K., Wang H.Y. (2009). Berberine inhibits hepatic stellate cell proliferation and prevents experimental liver fibrosis. Biol. Pharm. Bull..

[31] Zhang P., Qiang X., Zhang M., Ma D., Zhao Z., Zhou C., Liu X., Li R., Chen H., Zhang Y. (2015). Demethyleneberberine, a natural mitochondria-targeted antioxidant, inhibits mitochondrial dysfunction, oxidative stress, and steatosis in alcoholic liver disease mouse model. J. Pharmacol. Exp. Ther..

[32] Inagaki Y., Okazaki I. (2007). Emerging insights into transforming growth factor β Smad signal in hepatic fibrogenesis. Gut.

[33] Li J., Pan Y., Kan M.J., Xiao X.N., Wang Y.J., Guan F.Y., Zhang X.W., Chen L. (2014). Hepatoprotective effects of berberine on liver fibrosis via activation of AMP-activated protein kinase. Life Sci..

[34] Zuo F., Nakamura N., Akao T., Hattori M. (2006). Pharmacokinetics of berberine and its main metabolites in conventional and pseudo germ-free rats determined by liquid chromatography/ion trap mass spectrometry. Drug Metab. Dispos..

[35] Kheir M.M., Wang Y.G., Hua L., Hu J., Li L.L., Lei F., Dua L.J. (2010). Acute toxicity of berberine and its correlation with the blood concentration in mice. Food Chem. Toxicol..

[36] Bruck R., Aeed H., Shirin H., Matas Z., Zaide L., Avni Y., Halpern Z. (1999). The hydroxyl radical scavengers dimethylstioxide and dimethylthiourea protect rats against thioacetamide-induced f-ant hepatic failure. J. Heputol..

[37] Kim K.H., Bae J.-H., Cha S.-W., Han S.-S., Park K.H., Tae Cheon J. (2000). Role of metabolic activation by cytochrome P450 in thioacetamide-induced suppression of antibody response in male BALB/c mice. Toxicol. Lett..

[38] Buko V., Belonovskaya E., Naruta E., Lukivskaya O., Kanyuka O., Zhuk O., Kranc R., Stoika R., Sybirna N. (2015). Pituitary tumor transforming gene as a novel regulatory factor of liver fibrosis. Life Sci..

[39] Wu Y., Liu X., Zhou Q., Huang C., Meng X., Xu F., Li J. (2015). Silent information regulator 1 (SIRT1) ameliorates liver fibrosis via promoting activated stellate cell apoptosis and reversion. Toxicol. Appl. Pharmacol..

[40] Zhou D.J., Mu D., Jiang M.D., Zheng S.M., Zhang Y., He S., Weng M., Zeng W.Z. (2015). Hepatoprotective effect of juglone on dimethylnitrosamine-induced liver fibrosis and its effect on hepatic antioxidant defence and the expression levels of α-SMA and collagen III. Mol. Med. Rep..

[41] Ding H., Shi J.H., Wang Y., Guo J., Zhao J.H., Dong L. (2011). Neferine inhibits cultured hepatic stellate cell activation and facilitates apoptosis A possible molecular mechanism. Eur. J. Pharmacol..

[42] Kong D.S., Zhang F., Wei D.H., Zhu X.J., Zhang X.P., Chen L., Lu Y., Zheng S.Z. (2013). Paeonol inhibits hepatic fibrogenesis via disrupting nuclear factor-B pathway in activated stellate cells:in vivo and in vitro studies. J. Gastroen. Hepatol..

[43] Chen G., Wang Y.H., Li M.Q., Xu T.J., Wang X.L., Hong B., Niu Y.C. (2014). Curcumol induces HSC-T6 cell death through suppression of Bcl-2: Involvement of PI3K and NF-κB pathways. Eur. J. Pharm. Sci..

[44] Lang A., Schoonhoven R., Tuvia S., Brenner D.A., Rippe R.A. (2000). Nuclear factor κB in proliferation, activation, and apoptosis in rat hepatic stellate cells. J. Hepatol..

[45] Kanzler S., Lohse A.W., Keil A., Henninger J., Dienes H.P., Schirmacher P., Rose-John S., Zum Buschenfelde K.H.M., Blessing M. (1999). TGF-β 1 in liver fibrosis: An inducible transgenic mouse model to study liver fibrogenesis. Am. J. Physiol.-Gastrointest. Liver Physiol..

[46] Qiang X., Lulu X., Zhang M., Zhang P., Wang Y., Wang Y., Zhao Z., Chen H., Liu X., Zhang Y. (2016). Demethyleneberberine attenuates non-alcoholic fatty liver disease with activation of AMPK and inhibition of oxidative stress. Biochem. Biophys. Res. Commun..

[47] Nanji A.A., Mendenhall C., French S.W. (1989). Beef fat prevents alcoholic liver disease in the rat. Alcohol. Clin. Exp. Res..

[48] Ruwart M.J., Wilkinson K.E., Rush B.D., Vidmar T.J., Peters K.M., Henley K.S. (1989). The integrated value of serum procollagen III peptide over time predicts hepatic hydroxyproline content and stainable collagen in a model of dietary cirrhosis in the rat. Hepatology.

[49] Livak K.J., Schmittgen T.D. (2001). Analysis of relative gene expression data using real-time quantitative PCR and the 2^−ΔΔ*C*t^ method. Methods.

